# Symptomatic congenital Morgagni hernia presenting as a chest pain: a case report

**DOI:** 10.1186/s13256-019-2336-9

**Published:** 2020-01-18

**Authors:** Mujtaba Mohamed, Alsadiq Al-Hillan, Jay Shah, Eugene Zurkovsky, Arif Asif, Mohammad Hossain

**Affiliations:** Department of Medicine, Hackensack Meridian Health Jersey Shore University Medical Center, Neptune, NJ 07753 USA

**Keywords:** Morgagni hernia, Symptomatic, Chest pain

## Abstract

**Background:**

Morgagni hernia is a rare form of congenital diaphragmatic hernia with a prevalence of 2–3%. It occurs due to a defect on the anterior part of the diaphragm, which allows abdominal organs to penetrate into the thoracic cavity. This condition can be detected during fetal life by routine ultrasonography or late during adult life. Late diagnosis of this condition in adults is extremely rare. According to our literature search, only a few cases of symptomatic hernia in adults have been reported so far. Surgery provides definitive treatment for patients with Morgagni hernia; it is always recommended for symptomatic and asymptomatic adult patients to avoid future complications such as volvulus, small bowel obstruction, incarceration, or strangulation. We report a case of a patient who presented with chest pain due to newly diagnosed congenital diaphragmatic hernia.

**Case presentation:**

A 29-year-old unemployed white man with no significant past medical history or family history of coronary artery disease, who was a current smoker with a 1-pack-per-day history, presented to our hospital with a 1-month history of intermittent chest pain. His chest pain was localized to the right side with a pressure-like quality, moderate intensity 4–6/10, nonradiating, and relieved by standing up and worsened by lying flat. His pain was not associated with increase or decrease in activity level. The pain had progressively worsened, which prompted the patient to come to the emergency room. The patient was admitted for further evaluation. A chest x-ray showed a suspected loop of bowel on the right side of the chest. Subsequently, the patient underwent computed tomography of the chest, which revealed a 7-cm defect in the right hemidiaphragm with a large amount of intra-abdominal fat and a loop of the proximal transverse colon within the hernial sac. The patient was evaluated by a surgeon and eventually underwent laparoscopic repair of the diaphragmatic hernia with mesh repair. In follow-up, the patient’s symptoms resolved.

**Conclusion:**

Morgagni hernia is a rare form of congenital diaphragmatic hernia. It is commonly found either in the first few hours of life or in the antenatal period. It is less common in adults and is usually diagnosed accidentally in asymptomatic patients. Symptomatic adult cases are extremely rare. Respiratory symptoms are the most common presenting symptoms. The primary management for both symptomatic and incidentally discovered asymptomatic cases of Morgagni hernia is surgical correction. Various thoracic and abdominal surgical approaches have been described without a clear consensus on preference for operative repair technique.

## Introduction

Morgagni hernia (MH) was first described in 1769 by the Italian anatomist Giovanni Battista Morgagni as an anterior diaphragmatic hernia originating from the costosternal trigones, a triangular space located between the muscle fibers originating from the xiphisternum and the costal margin of the diaphragm and protruding into the central tendon [1]. The most common contents of the hernia sac include the omentum, followed by the colon, small bowel, stomach, and portions of the liver [2]. MH can occur on each side of the sternum; however, it is more common on the right side. Most cases are asymptomatic. In symptomatic cases, the most common presenting symptoms are cough and shortness of breath. Computed tomography (CT) is the most important tool for establishing the diagnosis. There are no guidelines for surgical treatment, owing to the rarity of cases. However, surgical repair is indicated in all cases to prevent strangulation. We present a rare case of symptomatic diaphragmatic hernia in a patient who presented with an unusual clinical presentation of chest pain and improved completely after laparoscopic surgical repair.

## Case presentation

**Fig. 1:**
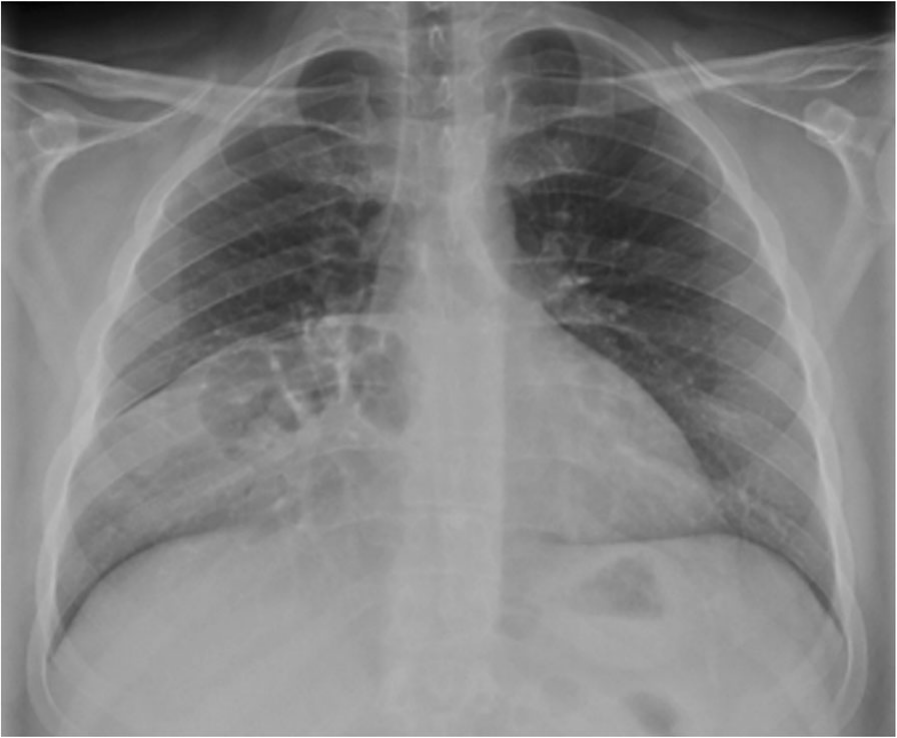
Chest x-ray showing posteroanterior view of bowel loops in the right pleural cavity

**Fig. 2:**
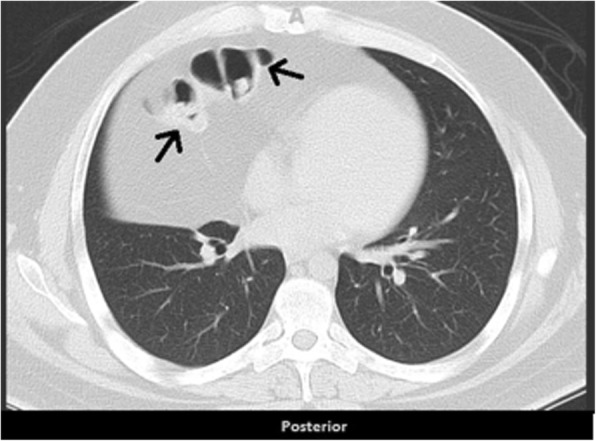
Computed tomographic scan (transverse section) showing bowel loops (*arrows*) and omentum herniating through the right side of the diaphragm

**Fig. 3:**
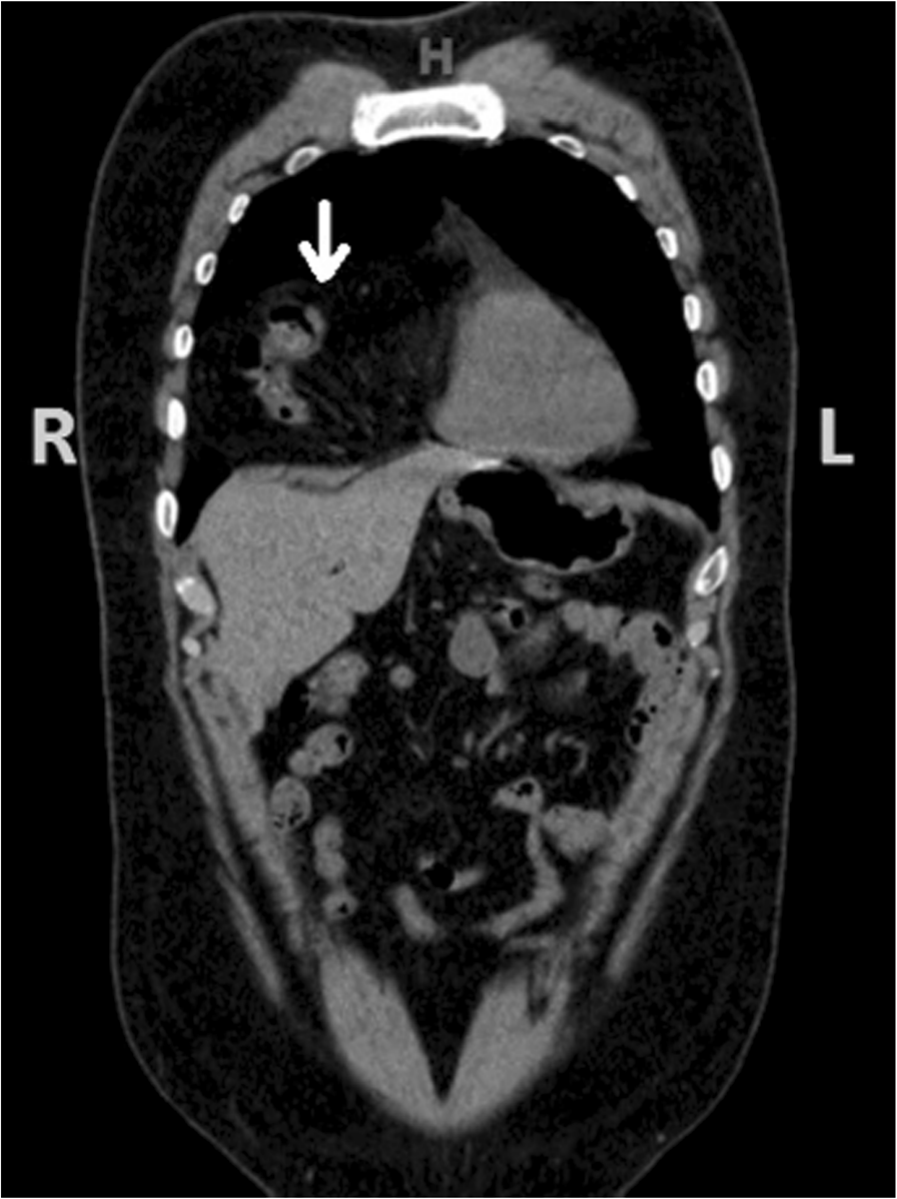
Computed tomographic scan (coronal section) with *arrow* showing bowel loops herniating through the right side of the diaphragm

**Fig. 4:**
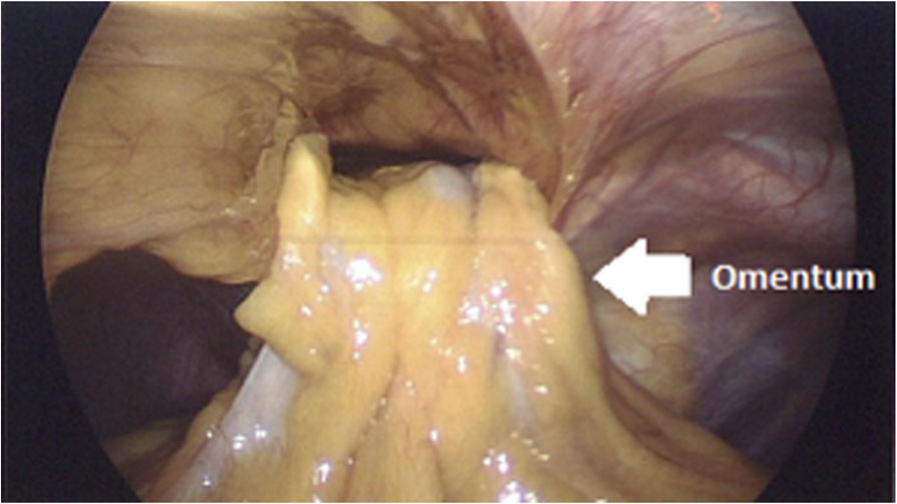
Computed tomographic scan (sagittal section) with *arrow* showing the diaphragmatic defect and herniated bowel loops in the pleural cavity

**Fig. 5:**
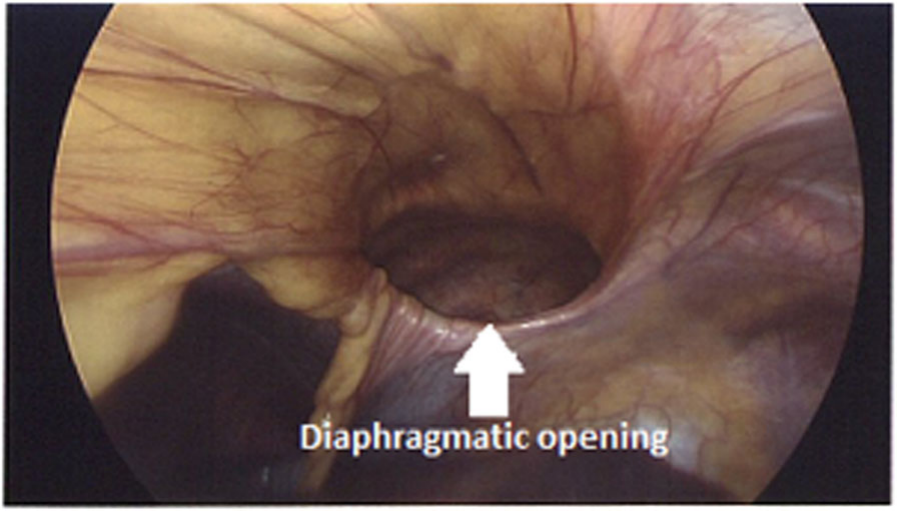
Defect opening in the diaphragm with peritoneum bulging into the pleural cavity

**Fig. 6:**
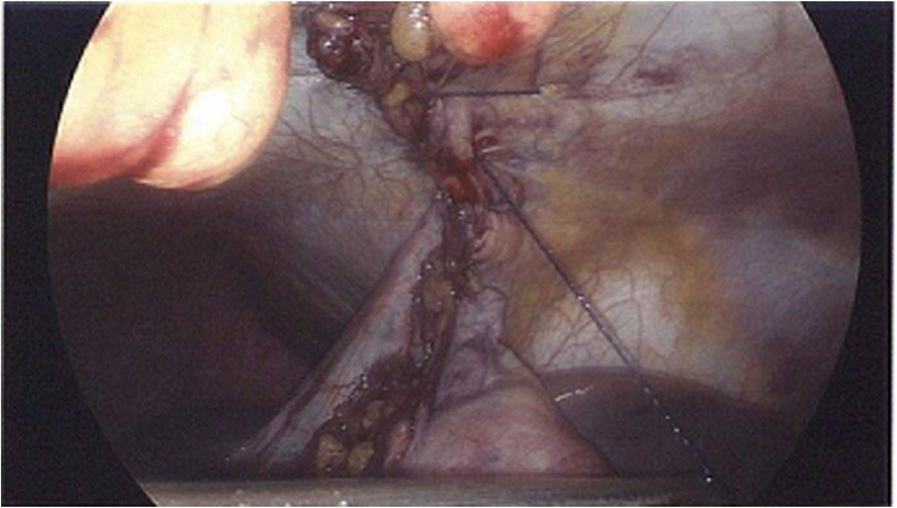
Repair of the diaphragmatic hernia with suturing

**Fig. 7:**
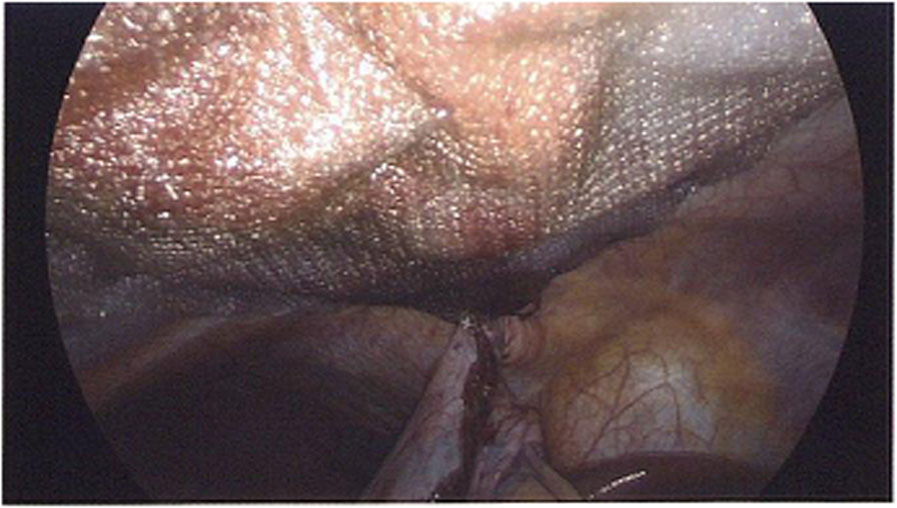
Placement of mesh during repair of diaphragmatic hernia

Our patient was a 29-year-old unemployed white man with no significant past medical history. He was a current smoker with a one-pack-per-day habit and a family history of coronary artery disease on his father’s side. He presented to the emergency room of our hospital with a 1-month history of intermittent chest pain. His chest pain was localized to the right side and was pressure-like, of moderate intensity 4–6/10, nonradiating, and relieved by standing up and worsened by lying flat, but otherwise it was not changed with increase or decrease in activity level. He had no associated palpitations, shortness of breath, dizziness, or lower extremity edema. He had been taking ibuprofen 500 mg orally as needed at home in an attempt to relieve his pain. Two days prior to this presentation, his chest pain became more constant with the same quality. On the day of admission, he developed difficulty in swallowing food. He experienced dysphagia (food stuck in the lower part of his esophagus); however, he had no associated nausea or vomiting. His physical examination revealed the following vital signs: blood pressure 144/75 mmHg and heart rate 72 beats per minute. Pulse oximetry showed his oxygenation was 99% on room air. Examination of his head, eyes, ears, nose, and throat revealed that his condition was normocephalic and atraumatic. His extraocular movements were intact. His pharynx was clear. His neck was supple without jugular vein distention. His chest wall was nontender. His lungs had clear breath sounds bilaterally without any evidence of wheezing, rales, or rhonchi. His cardiac examination revealed a regular rate and rhythm. His abdomen was soft and nontender with positive bowel sounds. His neurological examination revealed that he was alert and oriented to time, place, and person. His sensation was intact; he had no facial droop; and his pupils were equal and reactive to light and accommodation. His cranial nerves were intact. His power was 5/5 in all four extremities. His reflexes were intact. His complete blood count findings were as follows: white blood cell count 8300/μl (normal range 4500–11,000/μl), hemoglobin 14.4 mg/dl (12–16 mg/dl), hematocrit 41% (35–48%; 12–17.5 g/dl), and platelet count 273,000 (140,000–450,000/μl). His blood chemistry findings were as follows: sodium 139 mmol/ (normal range 135–145 mmol/L), potassium 4.1 mmol/dl (3.5–5.2 mmol/dl), chloride 106 mmol/L (96–110 mmol/L), CO_2_ 27 mmol/L (24–31 mmol/L), glucose 105 mg/dl (70–99 mg/dl), blood urea nitrogen 7 mg/dl (5–25 mg/dl), creatinine 0.72 mg/dl (0.44–1.0 mg/dl), aspartate aminotransferase 16 IU/L (10–42 IU/L), alanine aminotransferase 21 IU/L (10–60 IU/L), calcium 9.6 mg/dl (8.5–10.5 mg/dl), bilirubin 0.5 mg/dl (0.2–1.3 mg/dl), and lactate 1.0 mmol/dl (0.5–2.0 mmol/dl). His international normalized ratio was 1.14 (normal range 2–3 with conventional anticoagulation). The finding of his electrocardiogram (ECG) was negative for any ST changes. The patient’s chest x-ray showed a suspected loop of bowel on the right side of the chest (Fig. 1). Subsequently, the patient underwent CT of the chest, which showed a 7-cm defect in the right hemidiaphragm anteriorly with a large amount of intra-abdominal fat and a loop of proximal transverse colon within the hernial sac (diaphragmatic hernia of Morgagni). The herniated contents were located in the right pericardial location (Figs. 2, 3, and 4). A nasogastric tube was inserted to decompress the bowel. The patient was evaluated by a surgeon. Eventually, the patient underwent laparoscopic repair of his diaphragmatic hernia (Figs. 5, 6, and 7) with a successful outcome. His chest pain and dysphagia resolved completely. When he was seen 6 months later for follow-up, he was completely asymptomatic without any complications.

## Discussion

MH is a congenital diaphragmatic hernia. It is rare and comprises only about 2% of all diaphragmatic hernias [3]. We present a rare case of a young patient who presented with symptomatic diaphragmatic hernia with an unusual clinical picture of chest pain that improved completely after laparoscopic surgical repair. MH occurs due to an anteromedial diaphragmatic defect. Almost always, it occurs on the right side of the sternum (91%), which is the same side as in our patient; it occurs on the left side in only 5% of patients. Only 4% of the reported cases are bilateral. The defect results from a fusion failure of the diaphragm with the costal arches [2–6]. Sanford *et al.* reported that the average length of the diaphragmatic defect in the greatest dimension is 7.5 cm [6]. Our patient had a 7-cm defect in the right hemidiaphragm on the anterior part. Patients can be asymptomatic most of the time. Only a few rare symptomatic adult cases have been described [7]. Patients usually present in childhood with respiratory symptoms. In adults, MH can be misdiagnosed because it presents with nonspecific gastrointestinal and respiratory signs and symptoms. Respiratory symptoms are the most common presenting complaints, comprising about 34% of symptomatic cases [6]. In some cases, symptoms include cough, dyspnea, and chest pain. Our patient’s main presenting complaint was chest pain without respiratory symptoms. New-onset respiratory complaints in a formerly asymptomatic individual may be an early indication of progression of MH [8]. Abdominal pain can be due to incarceration or strangulation of the viscera [9, 10]. Pregnancy, trauma, obesity, chronic constipation, and chronic cough are common predisposing conditions contributing to the development of MH. Exercise and other types of exertion may also result in symptoms [11]. Women tend to present after the age of 50 years; men present earlier in life with complaints related to their hernia [8]. Li *et al.* reported that the most common abdominal organs found in the hernia sac are the colon and omentum, and less frequently the small bowel, stomach, and liver [3]. Our patient had transverse colon and omentum in his hernia sac. The presence of a hernia sac is associated with better outcomes, whereas thoracic herniation of the liver is associated with worse outcomes. In pediatric patients, comorbid conditions such as cardiac anomalies and major fetal defects, although more difficult to manage, had little effect on the outcome of the disease itself [12]. Although MH can be suspected on the basis of chest x-ray for workup for unexplained respiratory symptoms, CT of the chest and abdomen remains the modality of choice to confirm the diagnosis. CT is the most sensitive diagnostic tool because it provides anatomical details of hernia contents and its complications [13]. The most feared complication of MH is strangulation. On very rare occasions, gastric volvulus with small intestine diverticulosis can occur with MH [14]. Even if a patient is asymptomatic, surgical repair of MH is always indicated because of the risk of strangulation of hernia contents [3]. Surgical correction is the only established management for MH; however, because of the rarity of this pathology, there are currently no widely accepted guidelines on a standardized surgical technique in the literature [6]. The variety of surgical techniques currently available include open abdominal approaches via laparotomy; open thoracic approaches via median sternotomy or thoracotomy; and minimally invasive techniques, including laparoscopy and thoracoscopy. There are various advantages and disadvantages associated with each approach in the repair of MH [6]. The transabdominal approach is preferred for complicated cases in which bilateral hernias or those with dense intra-abdominal adhesions are suspected. In addition, if the diagnosis of MH is uncertain, this approach is beneficial because it provides the capacity for a complete inspection of the abdominal cavity [15]. Laparotomy is the most common approach for MH repair and is often used in emergent cases, especially when a patient presents with respiratory insufficiency or bowel obstruction [16]. The disadvantages of this approach are patient concerns regarding increased recovery time, cosmesis, and wound complications, thus requiring this technique to be considered only when other minimally invasive techniques are unavailable or inappropriate [16]. A transthoracic approach is used for large right-sided MH. It enables easier dissection of the hernia sac off the pleural and mediastinal structures with good visualization of the operative field [17]. This approach provides an effective repair of the hernia defect with minimal recurrence [18]. Limiting factors include possible postoperative intestinal obstruction, the risk of missing a bilateral hernia when present, and suboptimal access for the removal of the hernia sac [2]. This approach includes a median sternotomy and thoracotomy. Minimally invasive surgery in laparoscopy carries the shortest recovery time, offering almost immediate return to normal activities and diet by 3 days in a majority of cases and with a complication rate as low as 5%, which makes it the most favored approach in uncomplicated cases. However, this method may prove suboptimal for complicated cases, because failure to reduce contents may necessitate open surgery [2]. The postsurgical recurrence rate of MH is very low, and the results are excellent.

Use of mesh for MH repair is controversial, and it is not indicated for all patients. According to a series of 36 patients undergoing laparotomy or thoracotomy, surgeons were able to perform successful repair in the entirety of their MH cases without the use of mesh and without recurrence [19]. Mesh repair should be considered when there has been considerable tissue loss or notable thinning of the diaphragm or when primary tissue repair is not possible [20]. Our patient’s defect was repaired by using mesh to reinforce the primary repair and decrease recurrence (Fig. 7). Complications that can arise from mesh fixation include intrathoracic adhesions to the heart, lungs, or diaphragm as well as possible diaphragmatic rupture. However, the risks of postoperative complications related to mesh repair have been reduced in the era of composite covered mesh materials.

## Conclusion

MH is the rarest form of congenital diaphragmatic hernia and is commonly found either in the first few hours of life or in the antenatal period. It is less common in adults and is mostly diagnosed accidentally in asymptomatic patients. Symptomatic adult cases are even rarer and have a wide variety of symptoms. Although rare, MH should be considered in a young adult with chest pain after other causes are excluded.
